# Human alveolar hydrogels promote morphological and transcriptional differentiation in iPSC-derived alveolar type 2 epithelial cells

**DOI:** 10.1038/s41598-023-37685-x

**Published:** 2023-07-25

**Authors:** Evan T. Hoffman, Juan J. Uriarte, Franziska E. Uhl, Korin Eckstrom, Alicia E. Tanneberger, Chloe Becker, Chloe Moulin, Loredana Asarian, Laertis Ikonomou, Darrell N. Kotton, Daniel J. Weiss

**Affiliations:** 1 Department of Medicine, Larner College of Medicine, University of Vermont, 149 Beaumont Avenue, Burlington, VT 05405 USA; 2 Department of Physiology, College of Medicine, University of Kentucky, Lexington, KY 40506 USA; 3 Department of Experimental Medical Science, Lund University, Lund, Sweden; 4 Wallenberg Centre for Molecular Medicine, Lund University, Lund, Sweden; 5 Department of Microbiology and Molecular Genetics, Larner College of Medicine, University of Vermont, Burlington, VT 05405 USA; 6 Department of Oral Biology, University of Buffalo, The State University of New York, Buffalo, NY 14260 USA; 7 Cell, Gene and Tissue Engineering Center, University at Buffalo, The State University of New York, Buffalo, NY USA; 8 Center for Regenerative Medicine, Boston University and Boston Medical Center, Boston, MA 02118 USA

**Keywords:** Cell proliferation, Differentiation, Pluripotency, Self-renewal, Stem-cell niche, Stem cells, Transdifferentiation, Biomedical engineering, Biomaterials

## Abstract

Alveolar type 2 epithelial cells (AT2s) derived from human induced pluripotent stem cells (iAT2s) have rapidly contributed to our understanding of AT2 function and disease. However, while iAT2s are primarily cultured in three-dimensional (3D) Matrigel, a matrix derived from cancerous mouse tissue, it is unclear how a physiologically relevant matrix will impact iAT2s phenotype. As extracellular matrix (ECM) is recognized as a vital component in directing cellular function and differentiation, we sought to derive hydrogels from decellularized human lung alveolar-enriched ECM (aECM) to provide an ex vivo model to characterize the role of physiologically relevant ECM on iAT2 phenotype. We demonstrate aECM hydrogels retain critical in situ ECM components, including structural and basement membrane proteins. While aECM hydrogels facilitate iAT2 proliferation and alveolosphere formation, a subset of iAT2s rapidly change morphology to thin and elongated ring-like cells. This morphological change correlates with upregulation of recently described iAT2-derived transitional cell state genetic markers. As such, we demonstrate a potentially underappreciated role of physiologically relevant aECM in iAT2 differentiation.

## Introduction

Alveolar type 2 epithelial cells (AT2s) are facultative progenitors critical to lung homeostasis and regeneration^1,2^. Under normal lung conditions, AT2s are primarily quiescent cells that secrete surfactant through lamellar bodies in order to reduce alveolar surface tension during respiration, while additionally maintaining low levels of cellular differentiation to alveolar type 1 epithelial cells (AT1s) to preserve the alveolar epithelium^3,4^. However, following injury to the alveolar epithelium, AT2s are capable of responding to extracellular biochemical and mechanical signals by increasing cellular proliferation and differentiation into AT1s through a recently described transitional cell state intermediate^5–9^. The necessity of alveolar regeneration by AT2s is highlighted in several detrimental lungs conditions, notably pulmonary fibrosis, wherein dysfunctional AT2s stalled in a transitional cell state promote disease progression^8,10–12^.

Despite the widely acknowledged importance of AT2s in normal lung function, there have classically been significant difficulties in studying AT2 cellular biology and pathology in vitro. In particular, AT2s are challenging to maintain in traditional two-dimensional (2D) culture conditions in which observed cellular morphology and gene expression are skewed towards an AT1-like phenotype^13,14^. As such, significant effort over the past decade has been directed towards developing three-dimensional (3D) culture models for primary human AT2s^15,16^, as well as AT2s derived from human induced pluripotent stem cells (iAT2s)^17–20^. Methods for deriving iAT2s that recapitulate primary AT2 transcriptome and function (i.e. surfactant secretion through lamellar bodies) in 3D culture have now provided a reliable in vitro system for modeling AT2 function, maturation, and disease or infection^17,19,21–24^. Importantly, while initial studies on independently cultured iAT2s provided no evidence of differentiation towards AT1-like cells, several groups have recently demonstrated that 3D co-culture of iAT2s with fibroblasts promotes the emergence of a subset that of iAT2s that resemble both transitional^25^ and AT1-like^26^ cell states. Within these studies, co-cultured fibroblasts were characterized as expressing both physiologically relevant developmental growth factors and extracellular matrix (ECM) genes, suggesting an alteration in the surrounding extracellular environment^26^. And while the impact of fibroblast-derived developmental and growth factors on AT2 maturation and differentiation have been well described in vivo^27,28^, as well as using in vitro modeling^26,29^, far less is understood about the biochemical and mechanical impact of ECM on such processes, which have been shown to direct cellular differentiation in a variety of other tissue settings^30,31^.

To date, 3D cultures of independent iAT2s, as well as iAT2-fibroblast co-cultures, have primarily relied on Matrigel, an ECM derived from cancerous mouse tissue commonly utilized for stem and cancer cell proliferation^32^. While Matrigel provides an accessible and practical matrix for stem cell proliferation and maintenance, concerns about the origins, cellular contamination, and lot-to-lot variability of Matrigel provide a difficult obstacle in determining the role of ECM on cellular response^33^. To overcome this, several groups including our own have developed methodology for harnessing decellularized tissues as physiologically relevant biochemical and mechanical ECM platforms for ex vivo modeling in whole tissues^34–38^, as well as hydrogels for 3D modeling^39–46^. Recently, we have adapted previous protocols^47^, to include dissected decellularized lungs from patients with a normal lung history, idiopathic pulmonary fibrosis (IPF), or chronic obstructive pulmonary disease (COPD) in order to characterize proteomic differences between different regions of the lung, including alveolar-enriched ECM (aECM)^48^. While we found that aECM possessed an array of structural and basement membrane associated proteins, including a range of collagens, laminins, and proteoglycans, this study did not include the development of aECM as an ex vivo modeling system.

Herein, we describe the formation of aECM hydrogels from patients with a normal lung history as a physiologically relevant matrix for the 3D culture of iAT2s. Using mass spectrometry, we demonstrate that the hydrogel formation process (i.e. pepsin digestion) results in the enrichment of select ECM proteins, including fibrillar collagens and fibrillin-1 (FBN1), similar to a recent study in decellularized porcine brain hydrogels^45^, highlighting a commonly overlooked limitation of hydrogel formation on final ECM protein composition. Further, we show that aECM hydrogels may be formed at differing stiffnesses dependent on aECM protein concentration, allowing for more precise control of mechanical strain on iAT2s, a known factor impacting AT2 proliferation and differentiation through Hippo signaling and other pathways^49,50^. We demonstrate that aECM hydrogels are capable of facilitating iAT2 proliferation and spheroid formation (i.e. alveolospheres) similar to Matrigel cultures is described. Finally, we additionally characterize the elongation of a subset of iAT2s in aECM hydrogels, reminiscent of recently described human AT1-like cells in 3D culture^16^, which correlates with the upregulation of transitional cell state and AT1 genetic markers, recently described in iAT2-fibroblast co-culture^25,26^. Taken together, this study outlines the prospective role of physiologically relevant in situ ECM as a potential factor in iAT2 differentiation.

## Results

### Preparation of aECM hydrogels from decellularized human lungs

**Figure 1:**
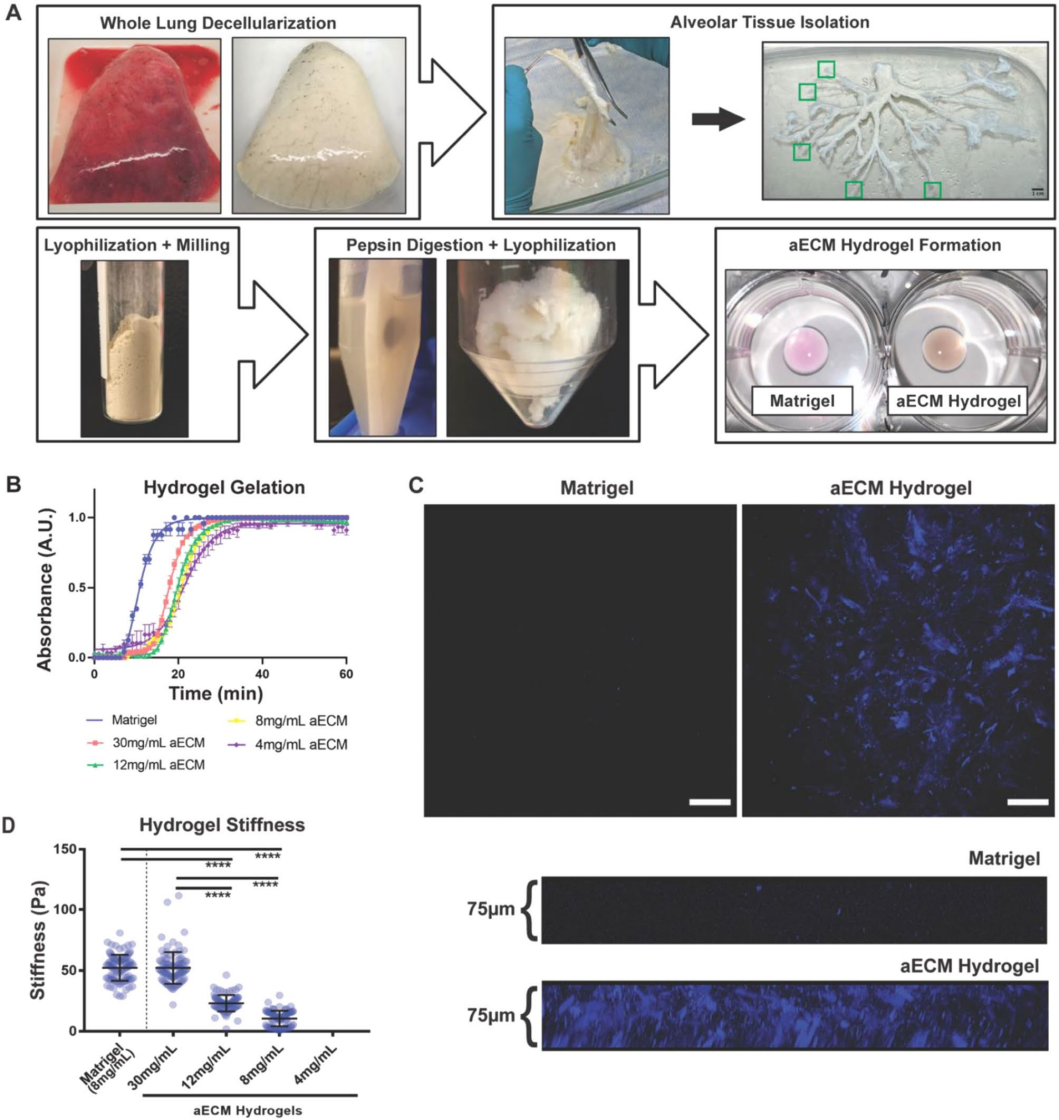
aECM powder is amenable to fibrous hydrogel formation. (**A**) Schematic demonstrating human lung decellularization, dissection, aECM powder derivation, and aECM hydrogel formation. Green boxes indicate distal tips of airways collected as the source of the aECM. Scale bar 1 cm. (**B**) Gelation curves of Matrigel and a range of aECM hydrogel protein concentrations (4, 8, 12, and 30 mg/mL aECM). Data points indicate mean ± SEM (n = 3) at each time point. (**C**) Second harmonic generation microscopy imaging depicting fibrillar ECM (i.e. COL1 and COL3) within aECM but not Matrigel hydrogels, scale bar 100 μm, as well as 75 μm cross-sectional z-stack images. (**D**) Matrigel and aECM hydrogel (4, 8, 12, and 30 mg/mL) stiffness (Pa) as measured by atomic force microscopy, mean ± SEM (n = 96), ****p < 0.0001.

To develop a physiologically relevant ex vivo culture model for iAT2s, as well as other applicable lung cells, we have designed a stepwise methodology for forming hydrogels derived from decellularized human lung alveolar-enriched ECM (aECM) (Fig. 1A). This methodology stems from previous works by our group demonstrating decellularized lung hydrogel formation for cell culture applications^39,40,51^, as well as the dissection of decellularized human lungs for region-specific proteomic assessment of the lung matrisome (i.e. ECM and ECM-associated proteins)^48^. Herein, aECM powder obtained and pooled from three individual patients with no history of lung disease was used as a starting material for aECM hydrogel formation (Supplemental Table 1). To form aECM hydrogels, aECM powder was digested with pepsin in HCl for 72 h, in accordance with previously published hydrogel formation protocols, in order to solubilize large polymerized ECM proteins including fibrillar collagens^42,43^. The solubilized aECM fraction was then lyophilized into cotton-like material that could be directly weighed and resuspended in culture media in order to form hydrogels with precise aECM protein densities (Fig. 1A). As iAT2s are typically cultured in Matrigel, we directly compared gelation potential and stiffnesses of hydrogels with a range of aECM protein densities (4, 8, 12, and 30 mg/mL) against 8 mg/mL Matrigel, a commonly used Matrigel protein density (Fig. 1B). We found that aECM hydrogels at all densities were capable of gelation over 20–30 min at 37 °C and a neutral pH compared to approximately 15–20 min required for Matrigel gelation (Fig. 1B).

Interestingly, while possessing a similar macroscopic phenotype to commercially available Matrigel (Fig. 1A), aECM hydrogels had a distinct organized fibrillar composition as evidenced by second harmonic multiphoton microscopy, indicating the presence of fibrillar collagens such as type-I and type-III collagen (COL1 and COL3) (Fig. 1C). In addition, we demonstrate that aECM protein density had a significant effect on hydrogel stiffness (Fig. 1D). In comparison the Matrigel, aECM hydrogels of the same protein concentration of 8 mg/mL were significantly less stiff (10.5 ± 0.8 Pa compared to 52.3 ± 1.1 Pa, respectively). However, by increasing the protein density to 30 mg/mL aECM hydrogels (52.3 ± 1.4 Pa) we were able to form a hydrogel of similar stiffness to Matrigel. Notably, 4 mg/mL aECM hydrogels were not stiff enough to be measured by atomic force microscopy (AFM) (Fig. 1D), despite being capable of gelation (Fig. 1C).

### Pepsin digestion significantly alters aECM protein composition

**Figure 2:**
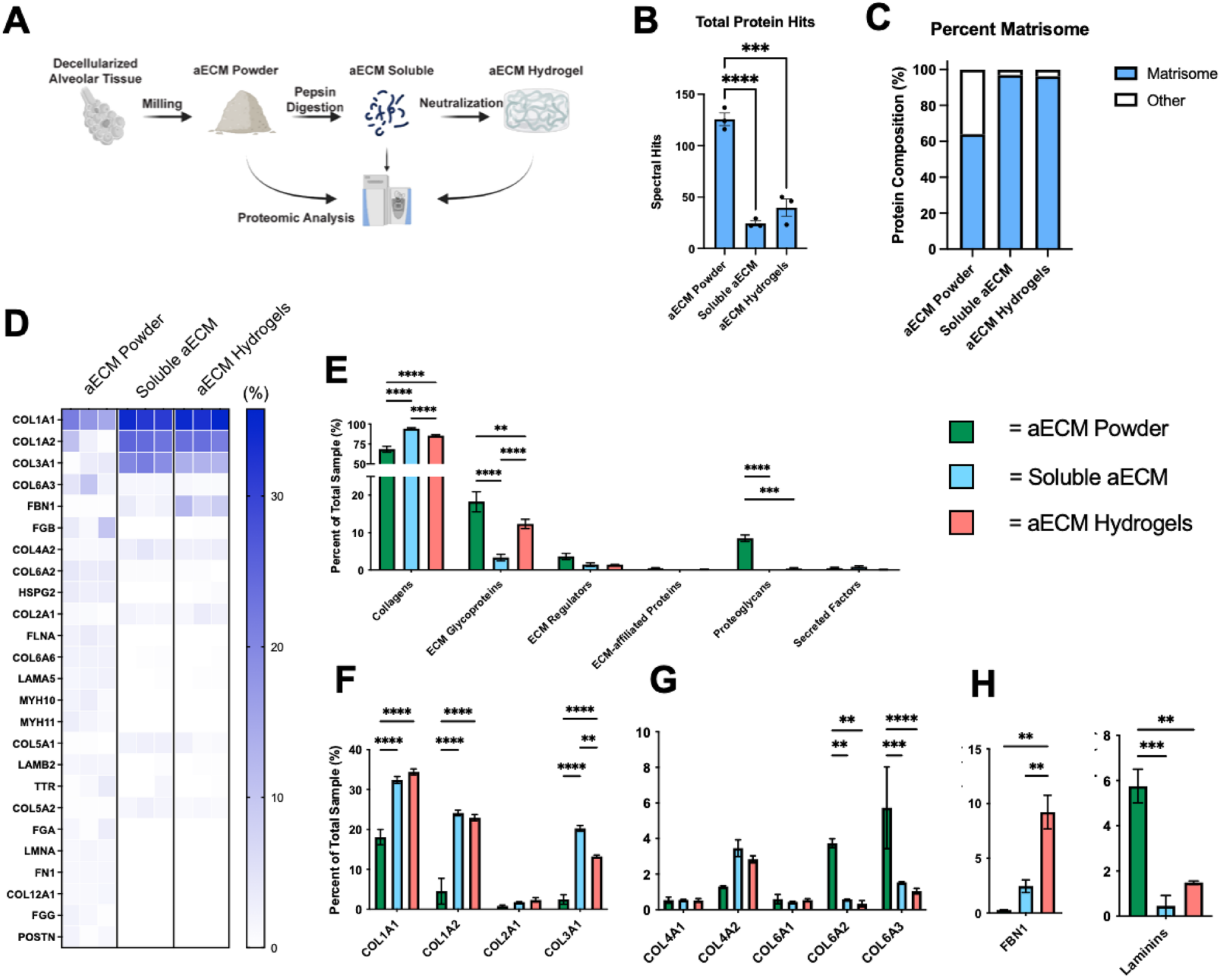
Pepsin digestion significantly alters aECM protein composition. (**A**) Schematic demonstrating critical points during the hydrogel formation process in which samples were taken for mass spectrometry. (**B**) Total unique proteins identified by mass spectrometry, mean ± SEM (n = 3), ***p < 0.001, ****p < 0.0001. (**C**) Percentage of total matrisome composition compared to total proteins. (**D**) Heatmap of Top 25 ECM Proteins in aECM powder samples (percent spectral hits compared to total sample spectral hits) compared to soluble aECM and aECM hydrogels. (**E**) Proportion of ECM protein types compared to total ECM composition, mean ± SEM (n = 3), **p < 0.01, ***p < 0.001, ****p < 0.0001. (**E**) Proportion of individual ECM proteins compared to total ECM composition categorized as (**F**) fibrillar collagens (**G**) other collagens, and (**H**) ECM glycoproteins, mean ± SEM (n = 3), **p < 0.01, ***p < 0.001, ****p < 0.0001.

We have previously demonstrated that the aECM powder utilized in these studies retains a large variety of native lung proteins, including basement-membrane associated type-VI and type-IV collagens (COL6 and COL4), fibrillar COL1 and COL3, laminins, and a range of ECM proteoglycans and glycoproteins^48^. As such, we determined that aECM powder, possessing a range of ECM proteins associated with alveolar tissue, may provide a more physiologically relevant culture model for iAT2s compared to Matrigel, which is largely composed of laminins, entactin, and COL4, as well as contaminating cellular proteins derived from cancerous mouse tissue. However, a recent proteomic study of hydrogels derived from decellularized porcine brain ECM demonstrated that pepsin digestion, a necessary step in the hydrogel formation process, resulted in a significant loss of native ECM proteins^45^. As such, we herein performed proteomic analysis of pepsin digested aECM (i.e. solubilized aECM) as well as fully formed aECM hydrogels to compare back to our previous dataset of decellularized aECM powder (Fig. 2A; Supplemental Table 2). We found that pepsin digestion resulted in a significant decrease in total unique proteins from aECM powder (~ 125) to soluble aECM (~ 24) and aECM hydrogels (~ 39) (Fig. 2B). Notably, despite the reduction in unique proteins following pepsin digestion, the remaining proteins were enriched for matrisome (ECM and ECM-associated proteins) as opposed to other proteins, such as cellular proteins retained through the decellularization process (Fig. 2C,D).

To further demonstrate the composition of aECM following pepsin digestion, we directly compared the relevant abundance of select ECM proteins in aECM powder, soluble aECM, and aECM hydrogels (Fig. 2D–H). As expected, given the necessary role of pepsin digestion in solubilizing fibrillar collagens previous to hydrogel formation, soluble aECM and aECM hydrogels possessed significantly higher proportion of overall collagen than aECM powder (Fig. 2E), including fibrillar COL1A1, COL1A2, and COL3A1 in particular (Fig. 2D,F)^41,52^. Further, soluble aECM also possessed a significantly higher proportion of overall collagens than aECM hydrogels, likely explained by the reassembly of insoluble fibrillar collagen during gelation, a process which may impair protein isolation methods during mass spectrometry preparation. Similar to a previous finding in decellularized porcine brain ECM, we demonstrate that fibrillin-1 (FBN1), a microfibrillar ECM protein involved in elastin formation, is enriched in soluble aECM and aECM hydrogels, suggesting dissociation of insoluble elastic fibers during the pepsin digestion process (Fig. 2D,H)^45^. In addition we found that pepsin digestion and hydrogel formation resulted in retention of COL4 (Fig. 2D,G), while other basement-membrane associated proteins (COL6 and laminins) were significantly reduced (Fig. 2D,G,H) or no longer detectable by mass spectrometry (fibronectin; FN1) (Fig. 2D).

In concordance with our previous report comparing proteomic signatures of individual regions and disease states of decellularized lungs, we found that the method of spectral analysis had a significant impact on our final results^48^. In particular, we found that the inclusion of dynamic hydroxyproline modifications during spectral analysis (Fig. 2; Supplemental Table 2) strongly favored the identification of fibrillar collagens, while absence of this modification resulted in increased detection of other significant ECM protein types, including ECM glycoproteins and ECM regulators (Supplemental Fig. 1; Supplemental Table 3). Importantly, we observed that the admittance of hydroxyproline modifications during our analysis resulted in increased identifications of overall laminins, suggesting an enrichment of laminins in aECM hydrogels (Supplemental Fig. 1G).

### aECM hydrogels facilitate iAT2 alveolospheres and a subset of elongated iAT2s

**Figure 3:**
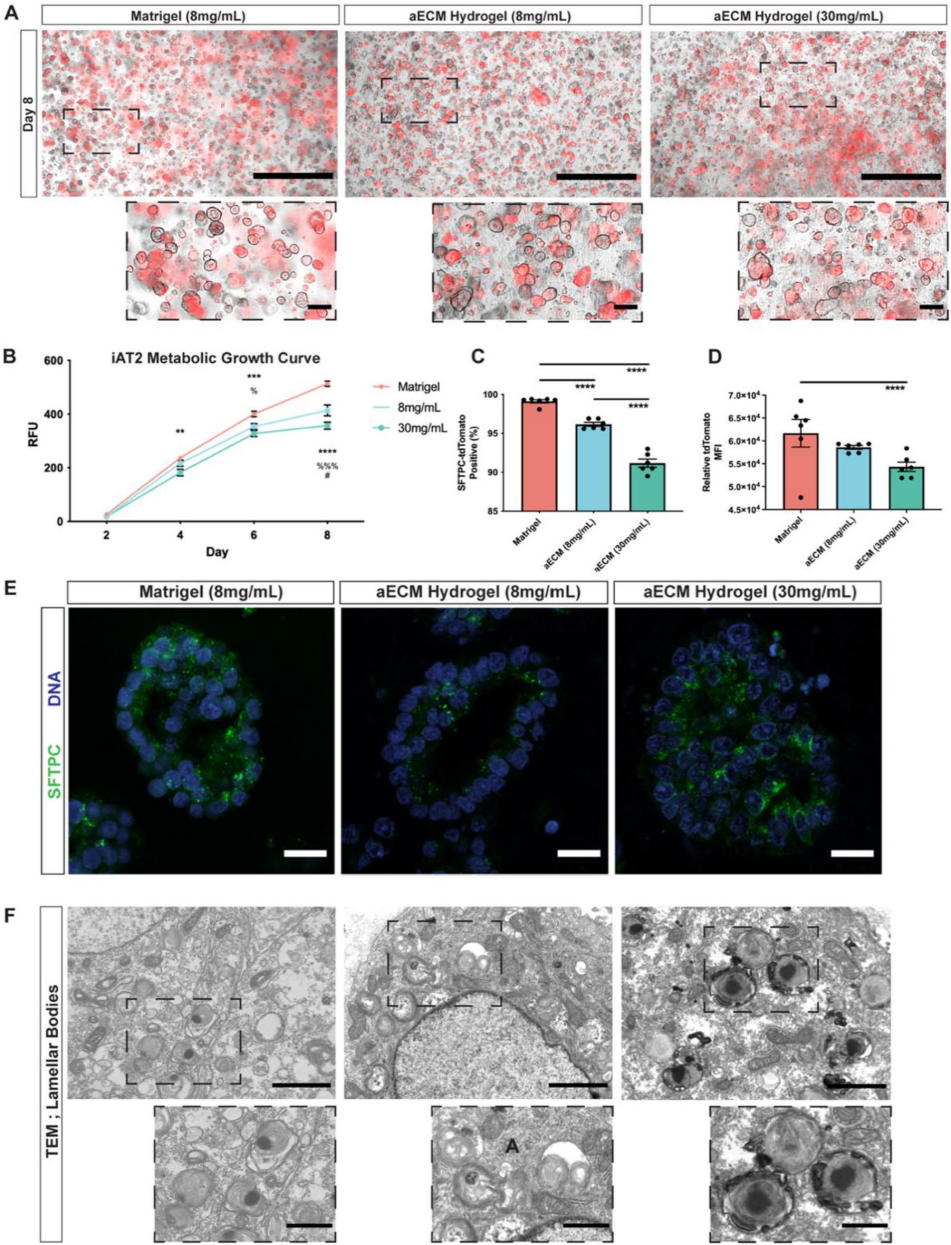
aECM hydrogels facilitate iAT2 proliferation and lamellar body formation. (**A**) Representative fluorescent imaging of iAT2 alveolosphere formation after 8 days in culture of Matrigel (8 mg/mL) or aECM hydrogels (8 mg/mL or 30 mg/mL) depicting brightfield and endogenous SFTPC-tdTomato fluorescence. Scale bar 1 mm. Insert scale bar 100 μm. (**B**) PrestoBlue metabolic growth curve of iAT2s represented in relative fluorescent units (RFUs), mean ± SEM, **p < 0.01, ***p < 0.001, ****p < 0.0001 = Matrigel vs. 30 mg/mL aECM hydrogel, ^%^p < 0.05, ^%%%^p < 0.001 = Matrigel vs. 8 mg/mL aECM hydrogel, ^#^p < 0.05 = 8 mg/mL hydrogel vs. 30 mg/mL hydrogel. (**C**,**D**) Percent positive and mean fluorescent intensity (MFI) of SFTPC-tdTomato in iAT2s by flow cytometry, ****p < 0.0001. (**E**) Representative immunohistochemical imaging of pro-SFTPC positive iAT2 alveolospheres. Scale bar 50 μm. (**F**) Representative transmission electron microscopy (TEM) of intracellular lamellar bodies in iAT2s. Scale bar 2 μm. Inset scale bar 1 μm.

To determine the impact of aECM hydrogels on iAT2 proliferation and phenotype, iAT2s carrying a fluorescent reporter (tdTomato) for surfactant protein C (SFTPC) were cultured in Matrigel compared to 8 mg/mL and 30 mg/mL aECM hydrogels, to control for protein density and stiffness, respectively (Fig. 3A). iAT2s were cultured according to previously protocols, including seeding cells into hydrogels as a single cell suspension as well as culturing in a previously defined AT2 directed differentiation and maintenance media^17,19^. We demonstrate that iAT2s formed SFTPC-tdTomato^+^ alveolospheres in both aECM hydrogel conditions comparable to Matrigel (Fig. 3A). Further, utilizing metabolism as a readout for iAT2 proliferation, we demonstrate that iAT2s produce similar growth curves in aECM hydrogels compared to Matrigel, despite proliferating slower in both aECM hydrogel conditions on Day 6 and Day 8 in culture (Fig. 3B). Endpoint analysis of dissociated iAT2s by flow cytometry at Day 8 demonstrates that significantly fewer iAT2s in both 8 mg/mL aECM hydrogels (95.6–96.9%) and 30 mg/mL aECM hydrogels (89.5–93.0%) retain SFPTC-tdTomato expression, compared to in Matrigel culture (98.1–99.4%) (Fig. 3C, Supplemental Fig. 1). Additionally, of the SFTPC-tdTomato^+^ cells in each condition, iAT2s in 30 mg/mL aECM hydrogels had a decreased tdTomato mean fluorescence intensity, suggesting a lower expression of SFTPC within tdTomato^+^ iAT2s as compared to Matrigel (Fig. 3D). Further, analysis of iAT2 alveolospheres in aECM hydrogels showed that alveolospheres positively stained for pro-SFTPC on the apical side of iAT2s facing the lumen of alveolospheres, similar to Matrigel, as well as possessing lamellar bodies responsible for surfactant secretion (Fig. 3E,F).

**Figure 4:**
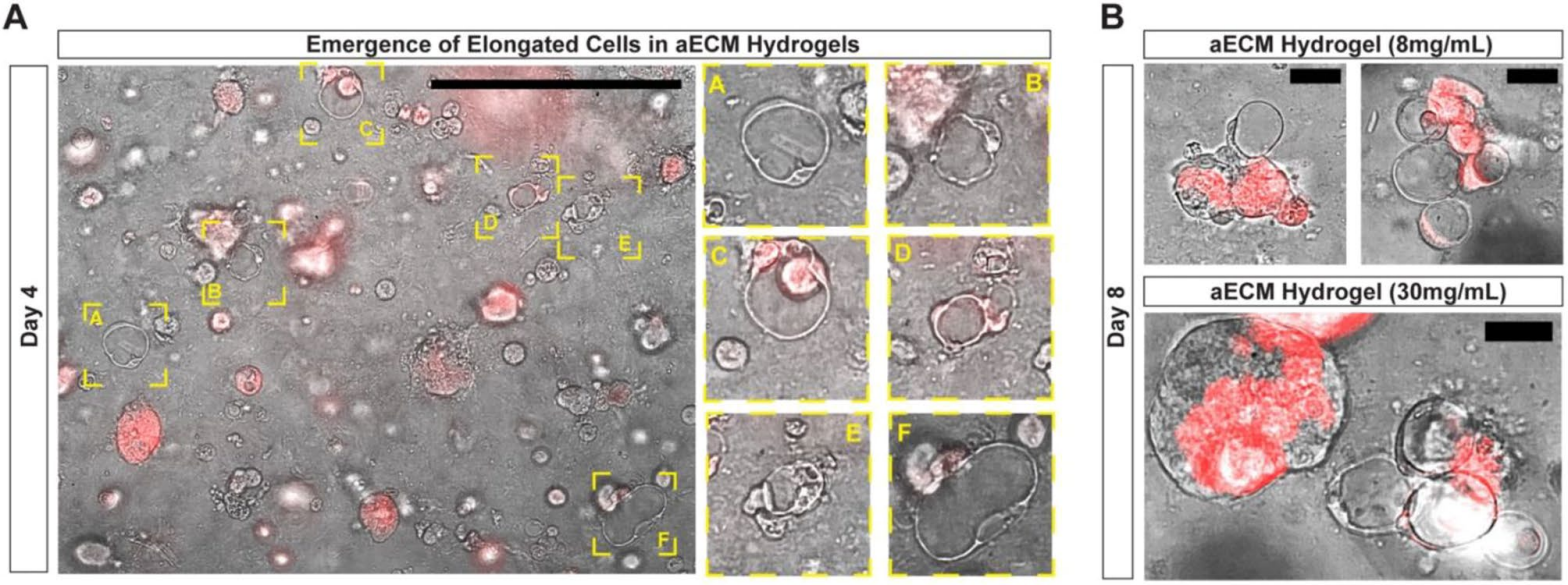
aECM hydrogels promote the emergence of a subset of elongated cells. (**A**) Representative fluorescent imaging depicting iAT2 alveolospheres and SFTPC-tdTomato positive and negative elongated iAT2s emerging after 4 days in aECM hydrogel culture. Scale bar 500 μm. (**B**) Retention of SFTPC-tdTomato positive and negative elongated iAT2s at 8 days in aECM hydrogel (8 mg/mL and 30 mg/mL) culture. Scale bar 50 μm.

Unexpectedly, by Day 4 in both aECM hydrogel conditions, we observed a subset of both tdTomato^+^ and tdTomato^−^ iAT2s with elongated cellular bodies, similar to recently described AT1-like cells in 3D culture (Fig. 4A)^16^. Elongated cells, not observed in Matrigel, were present in both aECM hydrogel conditions, and persisted throughout the duration of the culture period (Fig. 4A,B). Of particular interest, by Day 8 elongated cells were present in clusters, suggesting that distinct areas of aECM hydrogels promote elongation. Further, in rare instances elongated cells were present at the edge of iAT2 alveolospheres (Fig. 4B).

### Gene expression suggests aECM hydrogels promote transitional cell states in iAT2s

**Figure 5:**
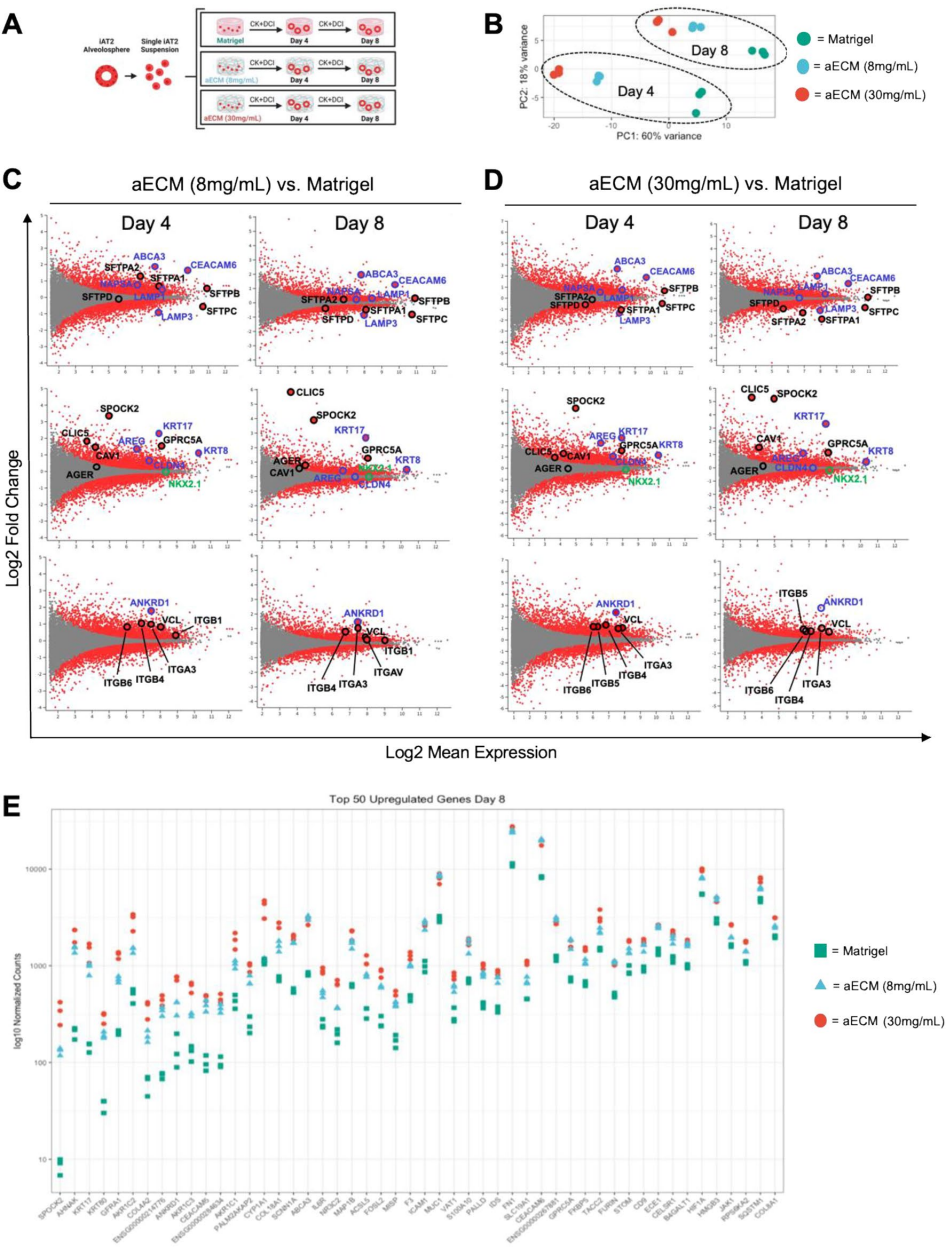
aECM hydrogels promote transitional cell state and AT1 gene expression. (**A**) Schematic representing iAT2 culture in Matrigel and aECM hydrogels for bulk RNA sequencing. (**B**) PCA plots demonstrating clustering of iAT2 gene expression within each condition. (**C**,**D**) MA plots demonstrating significant differentially expressed genes by adjusted p value (red dots), as well as non-significantly expressed genes (grey dots) at Day 4 and Day 8 between 8 mg/mL aECM hydrogels and Matrigel (**C**) or 30 mg/mL aECM hydrogels and Matrigel (**D**). Top row of MA (log ratio:mean average) plots labeled with surfactant (black) and lamellar-body associated (purple) AT2 markers. Middle row of MA plots labeled with AT1 (black), transitional cell state (purple), and lung-specific transcriptional factor (green). Bottom row of MA plots labeled with canonical Hippo signaling marker (purple) and focal adhesion marker and top for upregulated integrins (black). (**E**) Top 50 upregulated genes by log fold change at Day 8 between both aECM hydrogel conditions and Matrigel.

To further characterize the impact of aECM hydrogels on iAT2 behavior we next performed bulk RNA sequencing at Day 4 and Day 8 of iAT2s cultured in 8 mg/mL and 30 mg/mL aECM hydrogels as compared to Matrigel (Fig. 5A). We observed distinct transcriptomic differences in all conditions, with iAT2s in both 8 mg/mL and 30 mg/mL aECM hydrogels clustered more closely with each other by Principal Component Analysis (PCA) than with Matrigel culture at both Day 4 and Day 8 (Fig. 5B). In concordance with decreased expression of *SFTPC*-tdTomato in aECM hydrogel cultures, *SFTPC* gene expression was reduced in both aECM hydrogel conditions at both Day 4 and Day 8 (Fig. 5C,D). However, the expression of other surfactant genes were variable amongst 8 mg/mL and 30 mg/mL aECM hydrogels compared to Matrigel. We found that compared to Matrigel at Day 8, several surfactant genes in 8 mg/mL aECM hydrogels were downregulated (*SFTPA1*, *SFTPD*) and upregulated (*SFTPA2*, *SFTPB*), while in 30 mg/mL aECM hydrogels surfactant genes were downregulated (*SFTPA1*, *SFTPA2*, *SFTPD*) or unchanged (*SFTPB*). In addition, genes associated with lamellar body formation were generally upregulated (*ABCA3*, *CEAMCAM6*, *LAMP1*, *NAPSA*) at Day 4 and Day 8 in both aECM hydrogel conditions compared to Matrigel (Fig. 5C–E). Notably, we observed the expression level of the lung epithelial specific transcriptional factor (*NKX2.1*) in iAT2s cultured in 8 mg/mL aECM hydrogels and 30 mg/mL aECM hydrogels, in comparison to Matrigel, was unchanged or slightly downregulated, respectively (Fig. 5C,D).

Strikingly, RNA sequencing also demonstrated a significant upregulation of several genes associated with the recently defined (*KRT5*^−^/*KRT17*^+^) transitional cell state (*KRT17*, *KRT8*, *CLDN4*, *AREG*, *FN1*) in both aECM hydrogel conditions compared to Matrigel^5–8,25^, while basal cell marker KRT5 was not identified in any condition (Fig. 5B–E)^10^. By Day 8, there was also a significant upregulation of several recognized AT1 markers (*SPOCK2*, CLIC5, *GPRC5A*), despite little or no increase in another canonical AT1 marker (AGER) (Fig. 5C–E)^53–55^. Importantly, the increase in transitional cell markers and some, but not all, AT1 markers correlates with a recently described iAT2-derived transitional cell like state in fibroblast co-culture, suggesting a potential role of aECM hydrogels in producing a similar transitional cell state^25^.

In parallel, the canonical Hippo signaling marker, *ANKRD1*, was amongst the most upregulated genes in both aECM hydrogel conditions compared to Matrigel, suggesting the contribution of mechanical signaling to altered iAT2 genetic profile in aECM hydrogels (Fig. 5C–E). Further, expression of a variety of integrins, including β-1 integrin (*ITGB1*) and the focal adhesion protein vinculin (*VCL*) were significantly upregulated in aECM hydrogels compared to Matrigel, suggesting an increase in cell-ECM interactions (Fig. 5C,D). Interestingly, the altered gene expression profile in both aECM hydrogels shared a similar trend in upregulated (Fig. 5E) and downregulated (data not shown) gene expression compared to Matrigel, despite the stiffer 30 mg/mL aECM hydrogel condition leading to more robust gene expression difference (Fig. 5E).

### SFTPC-tdTomato- iAT2s primarily upregulate transitional cell state markers

**Figure 6:**
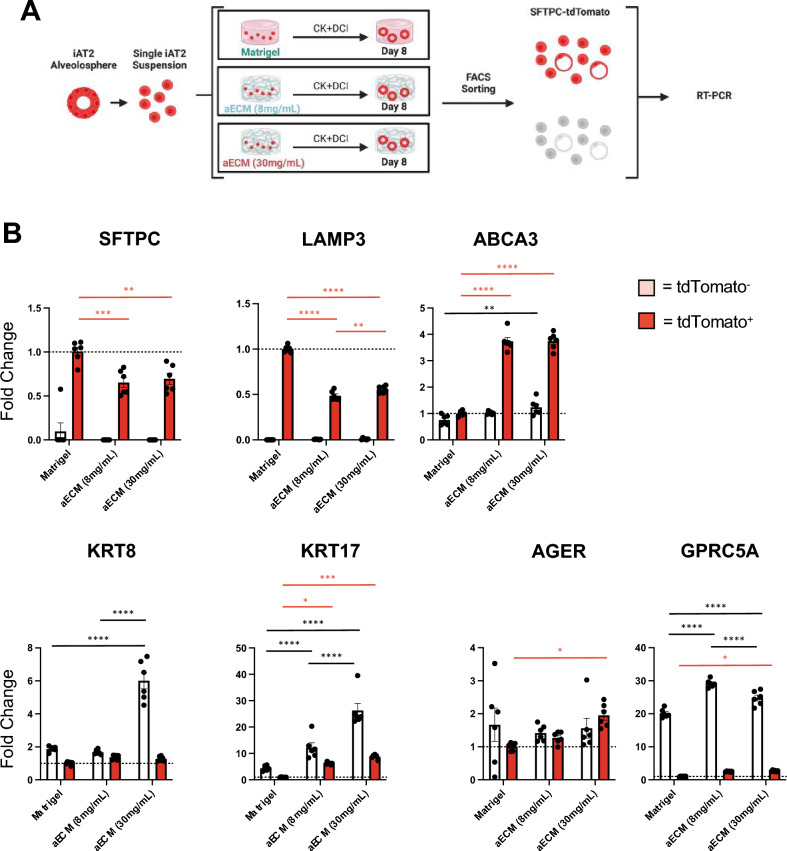
SFTPC-tdTomato^−^ iAT2s primarily upregulate transitional cell state markers. (**A**) Schematic representing FACS-sorting of Day 8 iAT2 cultures in each condition previous to RT-PCR. (**B**) Fold change differences of select genes by RT-PCR. Normalized to SFTPC-tdTomato^+^ iAT2s from Matrigel culture at Day 8, mean ± SEM (n = 6), *p < 0.05, **p < 0.01, ***p < 0.001, ****p < 0.0001. Red significance bars represent differences between SFTPC-tdTomato^+^ iAT2 gene expression and black significance bars represent differences between SFTPC-tdTomato^−^ iAT2 gene expression.

To further analyze the emergence of potential subpopulations of iAT2s in aECM hydrogels compared to Matrigel, we next characterized the expression of specific AT2, transitional cell state, and AT1-associated markers following sorting for SFTPC-tdTomato positive and negative cells from Day 8 cultures (Fig. 6A). We demonstrate efficient flow sorting due to negligible expression of SFTPC within tdTomato^−^ populations under any culture conditions (Fig. 6B). Trends of AT2 marker expression identified by bulk RNA sequencing were similar in tdTomato^+^ iAT2s, where there was a significant decrease in *SFTPC* and *LAMP3* in aECM hydrogel conditions compared to Matrigel, while there was a significant increase in the lamellar body associated marker *ABCA3* (Fig. 6B). With regards to recently described transitional state markers, we observed that the tdTomato^−^ population of iAT2s from 30 mg/mL aECM hydrogels expressed significantly more *KRT8* than 8 mg/mL aECM hydrogels or Matrigel, while tdTomato^−^ iAT2s from both aECM hydrogel conditions expressed significantly more *KRT17* than Matrigel. Interestingly, we also observed a significant upregulation of *KRT17* in tdTomato^+^ iAT2s in both aECM hydrogel conditions compared to Matrigel. Similar to bulk RNA sequencing results, we observed limited, if any, changes in expression of the canonical AT1 marker, *AGER* (Fig. 6B). However, we did observe significant upregulation of another AT1 marker, *GPRC5A*, in tdTomato^−^ iAT2s in both aECM hydrogel conditions as compared to Matrigel (Fig. 6B)^54^.

## Discussion

The derivation of iAT2s from human pluripotent stem cells utilizing directed differentiation protocols has provided a valuable in vitro 3D culture model for human AT2s that has rapidly contributed to our knowledge of AT2 function and disease^17,19,21–24^. However, while robust data exists describing the impact of soluble bioactive factors (i.e. developmental and growth factors) on iAT2 maturation and differentiation, far less is known about the impact of the surrounding ECM on such processes. Here, we sought to determine the impact of a physiologically relevant ECM on iAT2s utilizing human alveolar-enriched ECM (aECM) hydrogels as an ex vivo 3D culture model. We provide detailed proteomic analysis of aECM protein composition both before and after pepsin digestion, a standard processing step during hydrogel formation. As such, we highlight significant losses in native ECM protein composition due to pepsin digestion, an often overlooked limitation in previous ECM hydrogel studies. Further, in addition to facilitating iAT2 proliferation and alveolosphere formation, we demonstrate that aECM hydrogels promote morphological differentiation of a subset of iAT2s into thin elongated cells with a ring-like cellular body, similar to recently defined human AT1-like cells in 3D culture. The emergence of this elongated cell population correlates with the upregulation of (*KRT5*^*−*^*/KRT17*^+^) transitional cell state markers and some, but not all, AT1 markers recently described in iAT2s in fibroblast co-cultures. Taken together our studies suggest a potential role of relevant in situ ECM proteins in morphological and transcriptional differentiation of iAT2s.

While previous studies have not directly characterized the impact of ECM proteins on iAT2s, studies employing iAT2-fibroblast co-cultures have demonstrated using single-cell RNA sequencing that fibroblasts actively express ECM genes concurrent with the emergence of transitional cell state and AT1-like subpopulations of iAT2s, suggesting a potential role of ECM in iAT2 differentiation^25,26^. Further, a recent study has demonstrated the role of ECM-binding through β1-integrin as an important process in AT2 morphological and transcriptional differentiation^9^. Within this study, a mouse model of β1-integrin null AT2s found that, following injury to the alveolar epithelium, AT2 differentiation into elongated squamous AT1s was significantly inhibited^9^. This resulted in the accumulation of enlarged, round AT2s with transitional cell state genetic markers. While β1-integrin binds ECM targets, such as fibronectin, collagens, and laminins, this study suggests a potential role of ECM in AT2 differentiation.

The ECM is increasingly recognized as not only a structural scaffolding for tissues and organs, but also a critical driver of spatial cell development and function^30^. In particular, the ECM provides a range of biochemical and mechanical signals to resident cells that impact cell function including migration, proliferation, and differentiation. These signals may occur through direct ECM-binding to structural and bioactive ECM components, such as glycosaminoglycans, as well as through reservoirs of localized secreted extracellular factors, including Wnt signaling molecules and growth factors^31^. In addition to bioactive components, the ECM also provides distinct tissue-specific mechanical cues, including stiffness and mechanical stretch that drive cellular function through mechanosensory cellular pathways (i.e. Hippo and Erk kinase signaling)^56,57^.

To directly model the impact of a relevant in situ ECM on AT2 behavior, we sought to engineer aECM hydrogels, composed of ECM proteins derived from alveolar-enriched fractions of decellularized human lungs. This process builds upon previous decellularized lung hydrogel studies, including our own, that utilize entire decellularized lungs for ECM hydrogel formation, as opposed to specific anatomical lung regions^39,43,44,51^. Recently, we have further utilized dissected decellularized human lungs to describe proteomic ECM differences between individual anatomical regions (i.e. alveolar-enriched, airway, vasculature) in patients with a normal lung history, as well as patients with chronic obstructive pulmonary disease (COPD) or idiopathic pulmonary fibrosis (IPF)^48^. We found that aECM from patients with a normal lung history were enriched in ECM fibrillar collagens (COL1 and COL3), as well as basement membrane associated proteins including COL4 and COL6, laminins, and a range of proteoglycans. Here, we provide additional proteomic analysis of aECM after pepsin digestion and following aECM hydrogel formation. We demonstrate that pepsin digestion, which is necessary for solubilizing large collagen polymers prior to hydrogel formation, results in a significant decrease in native ECM proteins, while enriching for larger polymerized ECM proteins, namely COL1, COL3, and FBN1. Interestingly, the impact of pepsin digestion on native ECM protein composition has been overlooked in many ECM hydrogel studies, while only a single other study to our knowledge, utilizing hydrogels derived from decellularized porcine brain, has highlighted the limitation of pepsin digestion in the final ECM hydrogel composition^45^. Similar to the results presented here, pepsin digestion of porcine brain ECM resulted in the retention of only 18 unique ECM proteins identifiable by mass spectrometry, including primarily fibrillar collagens, FBN1, and laminins, while depleting other important ECM proteins including ECM glycoproteins such as aggrecan and versican. Our results, similarly demonstrate a reduction of unique native proteins from undigested aECM powder compared to pepsin-digested soluble aECM and aECM hydrogels. Notably, in comparison to porcine brain ECM hydrogels, aECM hydrogels were further enriched in FBN1, a protein involved in elastic microfibrils, which is likely due to the presence of insoluble elastin. While previous studies culturing organoids in ECM hydrogels from a variety of tissues demonstrate a potential role of the ECM in cellular maturation or differentiation (as reviewed in^46^), it is imperative to fully understand the final ECM hydrogel composition to evaluate cell-ECM interactions that may be driving these processes. Further, we demonstrate the importance of considering multiple methods of mass spectral analysis, namely the inclusion of hydroxylation of proline as a dynamic modification during this analysis. Similar to our recent proteomic study of regional decellularized lungs^48^, we found that the inclusion of hydroxyproline modifications favored the identification of fibrillar collagens, while decreasing identifications of other less abundant critical ECM proteins, including laminins. Taken together, we here highlight the significance of characterizing ECM composition at various stages of the hydrogel formation process to further inform future ECM hydrogels studies.

Upon culture of iAT2s in aECM hydrogels, we found that iAT2s in single cell suspension were capable of proliferating and forming alveolospheres similar to standardized Matrigel cultures. However, iAT2s in both conditions of aECM hydrogels (8 mg/mL and 30 mg/mL) proliferated at a reduced rate compared to standardized Matrigel culture and led to a decrease in the percentage of SFTPC-tdTomato^+^ iAT2s. This suggests that the aECM composition was causing a phenotypic change in a subpopulation of iAT2s despite being cultured in a previously defined directed differentiation media designed to promote AT2 phenotype retention^19^. Notably, we found that the stiffer 30 mg/mL aECM hydrogel conditions resulted in further SFTPC-tdTomato loss in iAT2s as compared to 8 mg/mL aECM hydrogels, suggesting a potential role in stiffness in loss of SFTPC expression and potential differentiation away from an AT2 phenotype.

We additionally observed the emergence of non-proliferative iAT2s at Day 4 that shared morphological similarities to recently defined AT1-like cells in 3D culture^16^. These elongated iAT2s had a thin, ring-like cellular body, variable SFTPC-tdTomato expression, and were present throughout the duration of the culture period, suggesting possible ongoing differentiation away from standard iAT2s in Matrigel culture. Interestingly, we commonly observed these elongated ring-like iAT2s in small clusters by Day 8 in both aECM hydrogel conditions. One potential explanation for this could be due to the proximity of iAT2s to dense collagen fibrils present throughout the aECM hydrogels, which will be further assessed in future studies.

Gene expression analysis by bulk RNA sequencing also suggested the emergence of iAT2 subpopulations in aECM hydrogels. We demonstrate that iAT2 culture in both aECM hydrogel conditions, in comparison to Matrigel, led to the upregulation of lamellar body-associated markers (*ABCA3*, *CEACAM6*, *NAPSA*, etc.), indicative of mature-like iAT2s, as well as markers recently described in an in iAT2 transitional cell state (*KRT17*, *KRT8*, *CLDN4*, *AREG*) and AT1-associated markers (*SPOCK2*, *GPRC5A*, *AHNAK*, *CLIC5*), suggesting diverse cell states. Interestingly, the upregulation of both transitional cell state markers, select AT1-associated markers, and absence of canonical AT1 marker AGER, has recently been described in an iAT2-derived transitional cell state, suggesting the emergence of similar transitional-like cell states in iAT2s cultured in aECM hydrogels^25^. As such, it is critical to further understand how aECM hydrogels impact iAT2s at a single cell level, and necessary follow up studies utilizing single cell RNA sequencing will be utilized to further characterize subpopulations of iAT2s in aECM hydrogel culture.

In addition, we demonstrate an upregulation of a constitutive Hippo signaling activation marker (*ANKRD1*) in iAT2s cultured in aECM hydrogels. Hippo signaling, a mechanically regulated signaling pathway commonly associated with the ECM, has been directly linked with AT2 differentiation^49,58^, and suggests a preliminary explanation of a mechanism promoting iAT2 differentiation in aECM hydrogels. Further, we found that *ANKRD1*, in parallel with transitional cell state markers, was increased in the stiffer 30 mg/mL aECM hydrogels as compared to 8 mg/mL aECM hydrogels, further suggesting a potential role in hydrogel stiffness in iAT2 morphological and transcriptional differentiation. Notably, however, while healthy lung tissue is considered to range from 1 to 5 kPa in stiffness, more recently advanced techniques that allow for modulation of decellularized lung hydrogel stiffness into the range of lung stiffness may provide additional information on the role of stiffness and mechanical strain on iAT2s morphological and transcriptomic differentiation^44,59^.

Taken together, our results indicate that aECM hydrogels provide a physiologically relevant matrix environment for iAT2 3D culture. We acknowledge that aECM hydrogels, similar to previously defined ECM hydrogels, do not possess the full range of native ECM proteins as previously described. However, aECM hydrogels possess a range of human collagens, laminins, as well as other ECM proteins that provide a more physiologically relevant matrix representing the in situ alveolar environment than does Matrigel. Further, utilizing aECM hydrogels, we provide evidence suggesting relevant ECM proteins are capable of driving iAT2s towards a differentiated cell state through a (KRT5^−^/KRT17^+^) transitional cell state intermediate. As such, this study highlights the role of specific ECM proteins, which are not digested by pepsin, to influence iAT2 cell states, and suggest similar proteins are involved in other ECM hydrogel studies involving cell-ECM interactions.

Our approach to deriving hydrogels from an individual anatomical region of decellularized human lungs provides a framework that may be applied to similar studies for other lung regions and disease conditions^48^. Moreover, the ability to manipulate hydrogels in vitro allows for future studies to address more targeted questions, such as the role of distinct ECM proteins on cell phenotype. This may include supplementing hydrogels with specific ECM proteins that are lost during the decellularization and pepsin digestion process^43^. In particular, future studies utilizing aECM hydrogels may provide a novel model to address the role of glycosaminoglycans on the alveolar epithelium, which have been suggested to impact AT2 differentiation through varying degrees of sulfation on the alveolar basement membrane^60,61^. This work would parallel with a previous study by our group which demonstrated that combinatorial supplementation of glycosaminoglycans and ECM-associated growth factors into hydrogels derived from whole decellularized human lungs had a significant impact on epithelial cell behavior^43^. Lastly, the ability to determine the role of normal versus diseased ECM, such as aECM derived from patients with end-stage IPF or COPD, may provide insight into the role of diseased ECM composition on aberrant cellular behavior and future therapeutic avenues.

## Materials and methods

### Human lung decellularization and region-specific isolation

Human lungs were acquired through UVM Autopsy Service in accordance with institutional guidelines and all subsequent experiments on human lungs were performed in accordance with institutionally approved protocols. Decellularization of the patient lungs utilized in this study was described and performed in a previous study according to our groups standardized decellularization protocols^47^ (Supplemental Table 1). In brief, whole lung lobes from patients with no history of lung disease (n = 3) were decellularized through sequential perfusion of detergent and enzyme solutions through airways and vasculature utilizing a peristaltic roller pump (Stockert Shiley, SOMA Technologies) at a 2 L/min rate^47^. Sequential 2L rinses included, 0.1% Triton-X 100 (Sigma), 2% sodium deoxycholate (SDC, Sigma), 1 M sodium chloride (NaCl, Sigma), 30 µg/mL DNase (Sigma)/1.3 mM MgSO_4_/2 mM CaCl_2_, 0.1%peracetic acid/4% ethanol (Sigma), and a DI wash. Confirmation of efficient decellularization for the lungs utilized in this study was performed previously^48^, including determination of < 50 ng/mg residual double-stranded DNA within decellularized lungs and the absence of DNA fragments by gel electrophoresis and nuclear staining by hematoxylin and eosin (H&E) staining, as previously described^47^.

Following validation of efficient lung decellularization, lung lobes were manually dissected to isolate specific anatomical regions including airways, vasculature, and alveolar-enriched regions^48^. Starting from the most proximal upper airways (i.e. trachea and large airways) and vasculature (which is positioned adjacent to large airways), decellularized lung lobes were dissected to the more distal regions of both the airway and vasculature trees capable of isolation. The most distal tips of the airways (< 1 mm) were isolated as alveolar-enriched ECM (aECM) from each patient and subsequently lyophilized and liquid nitrogen milled into a fine powder for storage at – 20 °C until future use.

### aECM solubilization and hydrogel formation

Given the interpatient proteomic similarities of individual patient lung aECM powder compositions^48^, aECM powder from all three patients was pooled previous to pepsin digestion and aECM hydrogel formation for all studies. Once pooled, aECM powder was resuspended at 10 mg/mL in a digestion solution consisting of 1 mg/mL pepsin (Sigma, #P7000) in 0.01 M HCl at pH of 2. Digestion was performed on a stir plate using fitted conical stir bars (V&P Scientific, #772FN) at room temperature for 72 h. Following digestion, the aECM solution was centrifuged at 1500 RCF for 5 min and the soluble fraction was removed from the insoluble pellet. This soluble fraction was utilized directly for some studies (mass spectrometry) or was continually processed for aECM hydrogel formation. Solubilized aECM was neutralized to a pH of 7 using sterile 0.1 M NaOH and frozen at − 80 °C. Frozen solubilized aECM was then lyophilized for 48 h to achieve a dry cotton-like material that could be stored at − 20 °C for future use. To form an aECM pre-gel solution, lyophilized aECM was weighed out and re-suspended in ice cold neutral pH culture media or PBS to a desired protein density, while remaining on ice with constant agitation for 2 h. aECM pre-gel solution was incubated at 37 °C for 1 h to allow for collagen reassembly and the formation of aECM hydrogels (i.e. gelation).

### Hydrogel gelation assessment

To assess gelation kinetics, hydrogel turbidity was assessed using spectrophotometry, as previously described^39,41,42^. 100μL of ice cold Matrigel (8 mg/mL) and aECM pre-gel solutions (4, 8, 12, and 30 mg/mL) in ice cold PBS were loaded into a 96-well plate (n = 3). Hydrogels were incubated at 37 °C for 1 h within a Synergy HT plate reader (BioTek) and absorbance values were measured at 405 nm once every 1 min. The absorbance values at each time point were normalized to PBS control wells, and the final normalized absorbance (NA) was calculated using the following equation, where R is the PBS-normalized value at each time point, Rmin is the lowest absorbance reading per well, and Rmax is the highest absorbance reading per well:

$$\mathrm{NA}=\left(\mathrm{R}-\mathrm{Rmin}\right)/(\mathrm{Rmax}-\mathrm{Rmin})$$

### Hydrogel stiffness assessment

For atomic force microscopy (AFM), 3D clear resin molds were designed using SolidWorks 2020^®^ and printed using on a Form 2 3D printer (FormLabs). The clear resin molds were adhered to tissue culture treated plastic (Falcon) using a spray-on adhesive. 100 μL of ice cold Matrigel or aECM hydrogel pre-gel solution (4, 8, 12, or 30 mg/mL) in PBS were loaded into the resin mold and allowed to solidify for 30 min (Matrigel) or 1 h (aECM hydrogels) at 37 °C. Matrigel and aECM hydrogels were overlaid with 50 µL of PBS and maintained at 37 °C during data acquisition to keep from drying and depolymerization. Samples were analyzed using an MFP-3D-BIOTEM AFM (Asylum Research). Hydrogels were indented with a 25 μm spherical plastic probe at a spring constant of approximately 10 pN/nm. 40 μm × 40 μm force maps of a were acquired and analyzed using Igor Pro (WaveMetrics)^62,63^.

### Hydrogel second harmonic imaging

To assess potential differences in fibrous ECM composition (i.e. fibrous collagen polymers), Matrigel and aECM hydrogel pre-gel solution in ice cold PBS (8 mg/mL) were plated at 50 μL on a 6-well plate and allowed to solidify for 30 min (Matrigel) or 1 h (aECM hydrogels) at 37 °C before being overlaid with PBS. Imaging was performed using a Zeiss LSM-7 MP and post-imaging analysis was performed using Fiji 2.1.0.

### Proteomics on soluble aECM and aECM hydrogel

Proteomics of soluble aECM and aECM hydrogels was performed according to our groups published mass spectrometry protocols for decellularized lung ECM^47^. Proteomic analysis of aECM powder was previously performed utilizing these same protocols^48^. In brief, soluble aECM was utilized to make 50 μL aECM hydrogels (8 mg/mL) in PBS as described above. Both soluble aECM and aECM hydrogels were mixed at a 1:1 ratio with 2 × mild Laemmli sample buffer (MLSC; 4% SDS, 125 mM Tris–HCl) and heated at 45 °C for 30 min and quantified by BCA assay (Thermo). Soluble aECM and aECM hydrogel samples were then run through an SDS-PAGE gel at 80 V for 10–15 min to remove detergent impurities. Protein bands were excised from the SDS-PAGE gel into small cubes (1 mm^3^) before destaining and tryptic in-gel digestion as previously described^47^.

Tryptic peptide samples were analyzed by mass spectrometry as previously described^48^. In brief, peptides were loading onto a fused silica microcapillary liquid chromatography (LC) column packed with C18 reversed-phase resin (1.8 µm 120A, UChrom C18). Peptides were introduced via a nano-electrospray ionization onto a Thermo Q-Exactive Plus (QE+) mass spectrometer (Thermo Electron, San Jose, CA) operating in a Higher-energy C-trap dissociation mode to obtain both MS and tandem MS (MS/MS) spectra. Mass spectrometry spectra were searched using the *Human* protein database (UniProt-proteome_UP000005640_human) with Proteome Discoverer 2.5 (Thermo Electron, San Jose, CA). Carboxymethylation of cysteines was set as a fixed modification; oxidation of methionine and hydroxylation of Proline (+ 15.995 Da) were set as dynamic modifications where described. Up to three missed tryptic peptide cleavages were considered, and three maximum dynamic modifications were allowed per peptide.

Result files from PD were analyzed using Scaffold (version Scaffold 5.1.1, Proteome Software Inc., Portland, OR), as previously described^48^. Proteins were assigned manually assigned to protein identifications groups (ECM, cytoplasm, cytoskeletal, nuclear, membrane-associated, or secreted) in accordance with previously defined lists published by Naba et al*.*^64^. ECM proteins were further classified as “matrisome” sub-groups (collagens, ECM glycoproteins, ECM regulators, ECM-affiliated proteins, proteoglycans, or secreted factors)^64^. ECM proteins were additionally defined as basement membrane associated proteins in accordance with Jayadev et al*.*^65^.

### Maintenance and culture of iAT2 alveolospheres

iAT2s were maintained in 3D Matrigel culture as alveolospheres, as previously described^17,19^. Single cell suspensions of iAT2s were seeded into 50 μL droplets of growth factor reduced Matrigel (Corning) at a cell density of 400–1000 iAT2s/µL and were cultured in 12-well plates (Corning). Matrigel droplets were allowed to solidify for 30 min at 37 °C before being overlaid with 1 mL of CK + DCI, in a previously described, fully-defined iAT2 directed differentiation and maintenance media (see Supplemental Table 4 for full reagent list)^17,19^. The first 2 days following cell seeding, CK + DCI media was additionally supplemented with 10 μM of the ROCK inhibitor Y-27632 (CK + DCI + Y; Tocris) to avoid detachment related apoptosis (I.e. anoikis). Media was refreshed every other day for approximately 2–3 weeks until iAT2 alveolospheres approached confluence. For passaging, Matrigel droplets were dissociated using 2 mg/mL Dispase (Gibco) for approximately 30 min to release alveolospheres. Isolated alveolospheres were washed with 10 mL of pre-warmed DMEM (Gibco) and centrifuged at 200 RCF for 5 min. For single-cell suspensions, alveolospheres were resuspended in 0.05% trypsin–EDTA (Gibco) and incubated for 15 min at 37 °C. After complete trypsinization, iAT2s were washed with DMEM containing 10% FBS (HyClone) and centrifuged at 300 RCF for 5 min. A single cell suspension of iAT2s were subsequently resuspended into an appropriate amount of Matrigel for continued routine passaging.

### Characterizing iAT2 proliferation in aECM hydrogels

For all experiments, single cell suspensions of iAT2s derived from Matrigel were resuspended in ice cold growth factor reduced Matrigel (8 mg/mL) or aECM pre-hydrogel solutions (8 or 30 mg/mL) in ice cold CK + DCI at a concentration of 1000 iAT2s/µL. Hydrogels were allowed to solidify for 30 min (Matrigel) or 1 h (aECM hydrogels) at 37ºC. Subsequently, hydrogels were overlaid with 1 mL of CK + DCI + Y for the first 2 days of culture and refreshed with CK + DCI every other day for the duration of the culture period. iAT2 proliferation, alveolosphere formation, and SFTPC-tdTomato expression was monitored using an IX70 Inverted Microscope (Olympus). Time-course analysis of metabolic output, as a proxy for cell proliferation, was characterized using Presto Blue Cell Viability Reagent (Thermo), according to the manufacturer’s protocol. In brief, Presto Blue Cell Viability Reagent was supplemented into cell culture media at 10% total volume and overlaid onto hydrogel droplets for 2 h at 37 °C. Subsequently, 100 µL of the culture media was transferred to 96-well plates (Grenier) and analyzed using a Synergy HT plate reader (BioTek).

### Flow cytometry for SFTPC-tdTomato expression

iAT2s were collected for flow cytometry analysis as previously described^17^. In brief, hydrogels were dissociated in either 2 mg/mL Dispase (Matrigel) or 1 mg/mL Liberase DL (aECM hydrogels; Roche) for 30 min at 37 °C to release alveolospheres. For a single-cell suspension, alveolospheres were incubated in 0.05% trypsin–EDTA for 15 min at 37 °C and washed in PBS/10%FBS as described above. Live cells were gated using a LIVE/DEAD Cell Imaging Kit (Thermo) and characterization of tdTomato-SFTPC expression was performed using a MACSQuant VYB Flow Cytometer (Miltenyi) and analyzed using the FlowJo_v10 software package.

### Whole hydrogel sectioning and immunohistochemistry

On Day 8, media was removed from Matrigel and aECM hydrogel cultures, which were then fixed within wells with 4% paraformaldehyde (PFA) for 30 min at room temperature. PFA was aspirated and hydrogels were washed in wells with PBS 3 times. Hydrogels were dehydrated in wells with 30% sucrose overnight at 4 °C. Whole hydrogels were embedded in OCT (Fisher) in 15 × 15 × 5mm cryomolds, flash frozen in liquid nitrogen, and stored at – 80 °C until further use. OCT-embedded hydrogels were sectioned (5 µm) using a Hacker-Bright OTF 5000 cryostat maintained at – 20 °C. Sectioned alveolospheres were blocked for 60 min with 10% donkey serum/5% BSA/0.1% Triton X-100 in PBS. Samples were washed with 5% BSA, and stained with 1:2000 anti-pro-SFTPC (Millipore #AB3786) in PBS/5%BSA overnight at 4 °C. Samples were washed in 5% BSA and incubated with 4 µg/mL AlexaFluor donkey anti-rabbit IgG-555 (Invitrogen #A31572) in PBS/5%BSA for 1 h at room temperature. Nuclei were stained with 10 µg/mL DAPI (Invitrogen #D1306) and slides were mounted using DAKO IF Mounting Media (#S3023). Images were obtained using a Nikon A1R-ER and post-imaging analysis was performed using Fiji 2.1.0.

### Electron microscopy of iAT2s

At Day 8, whole alveolospheres were harvested from Matrigel and aECM hydrogels as described above, and prepped for electron microscopy as previously described^19^. In brief, alveolospheres were fixed with 2.5% glutaraldehyde in 0.1 M cacodylate buffer for 3 h at room temperature. Samples were washed with 0.1 M cacodylate buffer, treated with 1% Tannic acid in 0.1 M cacodylate buffer for 5 m at room temperature, and washed again with 0.1 M cacodylate buffer, and treated with 1.5% osmium tetroxide in 0.1 M cacodylate buffer overnight at 4 °C. Samples were washed with 0.05 M Na Maleate buffer (pH of 5.2) and stained with 1.5% Uranyl acetate in 0.025 M Na Maleate buffer (pH of 6). Samples were then dehydrated with 70%, 80%, and 90% acetone on ice. Samples were then incubated in 100% acetone for 10 min at room temperature and propylene oxide for 15 min at room temperature; this process was repeated a second time. Samples were then embedded in Spurr’s Resin (EMS #14300), according to the manufacturers protocol. Spurr’s Resin was added to the dehydrating solution at 1:1 overnight, and again at 1:3 for 2 h, before 100% Spurr’s Resin was added to the samples and cured overnight at 60 °C. Embedded samples were sectioned (70 nm) and grids were subsequently stained with 4% aqueous uranyl acetate for 5 min at 60 °C and then lead citrate for 10 min at room temperature. Grids were imaged using a JEOL 1400 Transmission Electron Microscopy with an AMT XR611 high resolution 11 megapixel mid-mount ccd camera and post-imaging analysis was performed using Fiji 2.1.0.

### RNA sequencing of iAT2s

At Days 4 and 8, whole alveolospheres were retrieved from Matrigel and aECM hydrogels culturing with 2 mg/mL Dispase (Matrigel) and 1 mg/mL Liberase DL (aECM hydrogels) incubated for 30 min at 37 °C and washed with PBS as described above. The dry alveolosphere pellet was lysed using RLT Buffer (Qiagen). RNA was extracted using miRNeasy Cells Advanced Micro Kit (Qiagen), according to the manufacturers protocols, and RNA was quantified using a Qubit spectrofluorometer (Quantus). Sequencing libraries were prepared from total RNA samples using SMARTer Stranded Total RNA-Seq Kit v2 (Takara) and samples were sequencing using an Illumina HiSeq 1500. Raw reads were checked for quality using FastQC and subsequently trimmed of Illumina adaptors and poor quality bases (< Q20) with a minimum length of 35 base pairs using Trim Galore! v. 0.6.4. Trimmed reads were then mapped to GRCh38 with ALT contigs and padded N's removed, using STAR v. 2.7.4a. Duplicates were removed using the MarkDuplicates function of Picard, then counts were generated using HTSeq. Differential expression was performed using DESeq2. Raw counts were imported, adjusted for library size, and normalized with default methods, and a base filtering of > 0.1 was performed to remove low level or rare gene counts. Differential expression was tested using the interaction of dosage group * treatment day using a Wald test and a significance threshold of < 0.05 with Benjamini–Hochberg correction.

### FACS and RT-PCR

At Days 4 and 8, a single cell suspension was retrieved from Matrigel and aECM hydrogel cultures as described above. In brief, 2 mg/mL Dispase (Matrigel) and 1 mg/mL Liberase DL (aECM hydrogels) for 30 min at 37 °C to release alveolospheres, before retrieving a single cell suspension using 0.05% trypsin–EDTA for 15 min 37 °C. Single iAT2s were then washed in PBS/10%FBS. Samples were resuspended in 2%FBS in Hank’s Balanced Salt Solution (ThermoFisher) with 10 µM ROCK inhibitor (Y-27632) and 10 µM of Calcein blue (Thermo) for the identification of live cells and passed through a 40 µm cell strainer (Fisher). SFTPC-tdTomato^+^ and SFTPC-tdTomato^−^ iAT2s from each condition were separately collected using a BD FACSAria cell sorter with BD FACSDIVA v8 software. Following collection, a TaqMan Fast Advanced Cells-to-CT Kit (Thermo) was used to lyse cells and prepare samples for RT-PCR without the need for separate RNA purification or cDNA synthesis steps. TaqMan probes were utilized for primers, including a primer-limited 18S endogenous control for multiplexing (see full primer table in Supplemental Table 5). RT-PCR was conducted using a QuantStudio6 384-well program. Relative gene expression of each target gene was normalized to the multiplexed 18S control from each sample and was depicted as a fold change difference (2^(−∆∆CT)^), as compared to gene express from SFPTC-tdTomato^+^ iAT2s cultured in Matrigel.

### Statistical analyses

Figures depicting hydrogel gelation or stiffness, flow cytometry, or RT-PCR data are presented as mean and standard error of mean. Significance was determined using GraphPad Prism (Mac 9.3.1.) with ordinary one-way ANOVA and Tukey’s multiple comparison t-test, with threshold significance at p < 0.05. Proteomic analysis of individual proteins was performed using two-way ANOVA and Sidak’s multiple comparison’s test, with threshold significance at p < 0.05.

## Supplementary Information

Supplementary Figures.

Supplementary Legends.

Supplementary Tables.

## Data Availability

Proteomic data for aECM powder was previously published and available on MassIVE (#MSV000090771). Proteomic data for soluble aECM and aECM hydrogels is also available on MassIVE (#MSV000091470). RNA sequencing data generated in this study is available on GEO (GSE227546).
