# Supplementary material for: Human alveolar hydrogels promote morphological and transcriptional differentiation in iPSC-derived alveolar type 2 epithelial cells
Source: Sci Rep. 2023 Jul 25;13:12057. doi: 10.1038/s41598-023-37685-x (PMC10368739; doi:10.1038/s41598-023-37685-x)
Supplement: Supplementary file 1 — Supplementary Figures. [file 41598_2023_37685_MOESM1_ESM.pdf]

Supplemental Table 1. Patient Demographics. Patient demographic data corresponding to decellularized lungs utilized in this study.

Supplemental Table 2. Mass spectral data of aECM during hydrogel formation process. Mass spectral hits during three critical steps of the aECM hydrogel formation process including lyophilized aECM powder, soluble aECM following pepsin digestion, and final aECM hydrogels. Proteins are categorized by their subcellular location and matrisome-affiliation for further analysis.

Supplemental Table 3. Mass spectral data of aECM during hydrogel formation process excluding hydroxylation of proline as a dynamic modification. Secondary mass spectral analysis of the aECM powder, soluble aECM, and aECM hydrogel samples excluding hydroxylated proline as a dynamic modification during data analysis. Proteins are categorized by their subcellular location and matrisome-affiliation for further analysis.

Supplemental Table 4. Differentiation media components for iAT2s. Recipe for iAT2 differentiation maintenance media utilized for all cell culture experiments.

Supplemental Table 5. TaqMan probe list. List of TaqMan probes utilized for RT-PCR.
