# Supplementary material for: Human alveolar hydrogels promote morphological and transcriptional differentiation in iPSC-derived alveolar type 2 epithelial cells
Source: Sci Rep. 2023 Jul 25;13:12057. doi: 10.1038/s41598-023-37685-x (PMC10368739; doi:10.1038/s41598-023-37685-x)
Supplement: Supplementary file 2 — Supplementary Legends. [file 41598_2023_37685_MOESM2_ESM.pdf]

Supplemental Figure 1. Re-analysis of mass spectrometry analysis in the absence of hydroxyproline modifications.

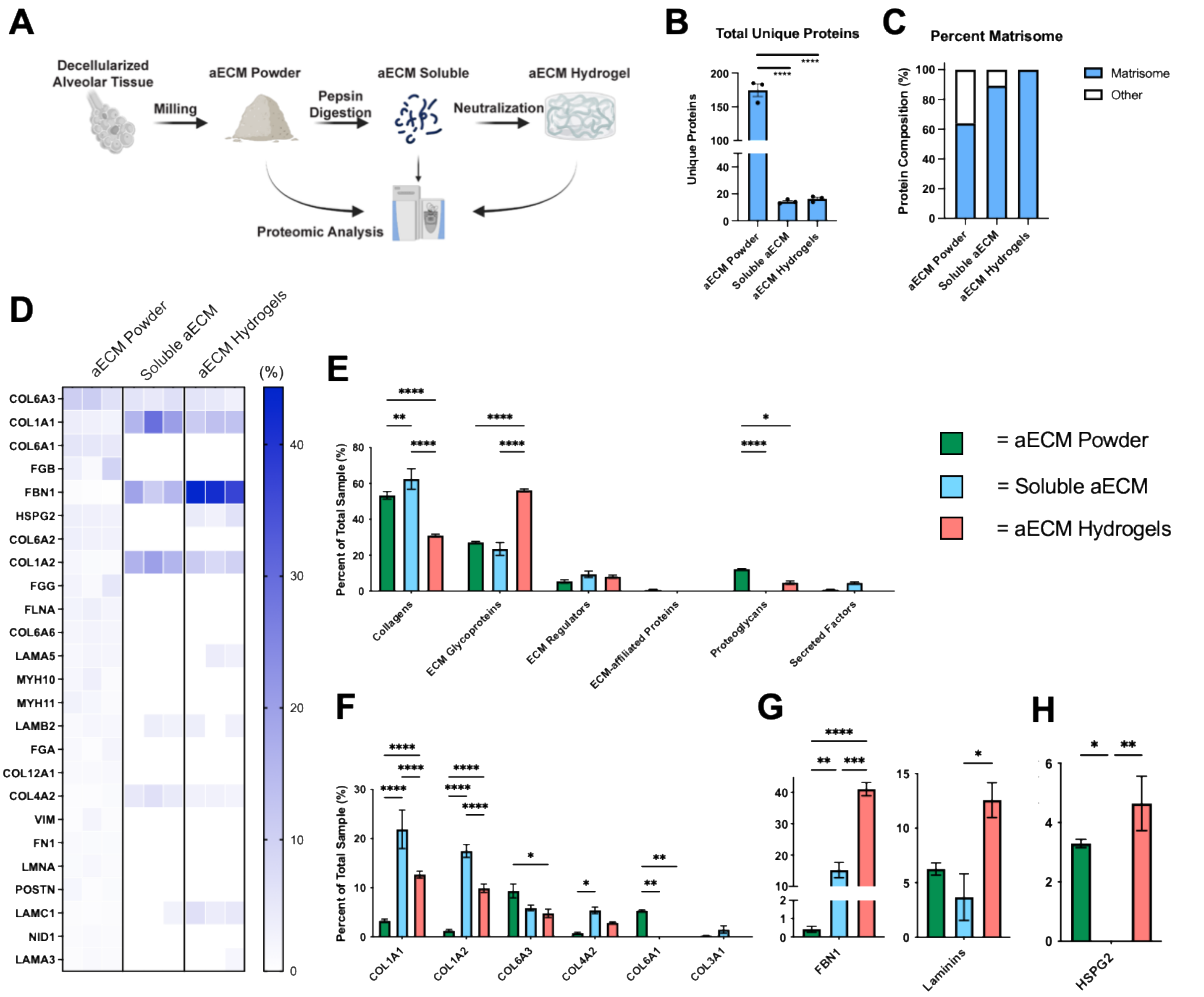

Supplemental Figure 1. Re-analysis of mass spectrometry analysis in the absence of hydroxyproline modifications. (A) Schematic demonstrating critical points during the hydrogel formation process in which samples were taken for mass spectrometry. (B) Total unique proteins identified by mass spectrometry, mean ± SEM (n=3), ***p<0.001, ****p<0.0001. (C) Percentage of total matrisome composition compared to total proteins. (D) Heatmap of Top 25 ECM Proteins in aECM powder samples (percent spectral hits compared to total sample spectral hits) compared to soluble aECM and aECM hydrogels. (E) Proportion of ECM protein types compared to total ECM composition, mean ± SEM (n=3), **p<0.05, **p<0.01, ***p<0.001,****p<0.0001. (E) Proportion of individual ECM proteins compared to total ECM composition categorized as (F) fibrillar collagens (G) ECM glycoproteins, and (H) HSPG2 proteogylcan, mean ± SEM (n=3), **p<0.05, **p<0.01, ***p<0.001,****p<0.0001.

Supplemental Figure 2. Gating strategy for tdTomato expression in iAT2s.

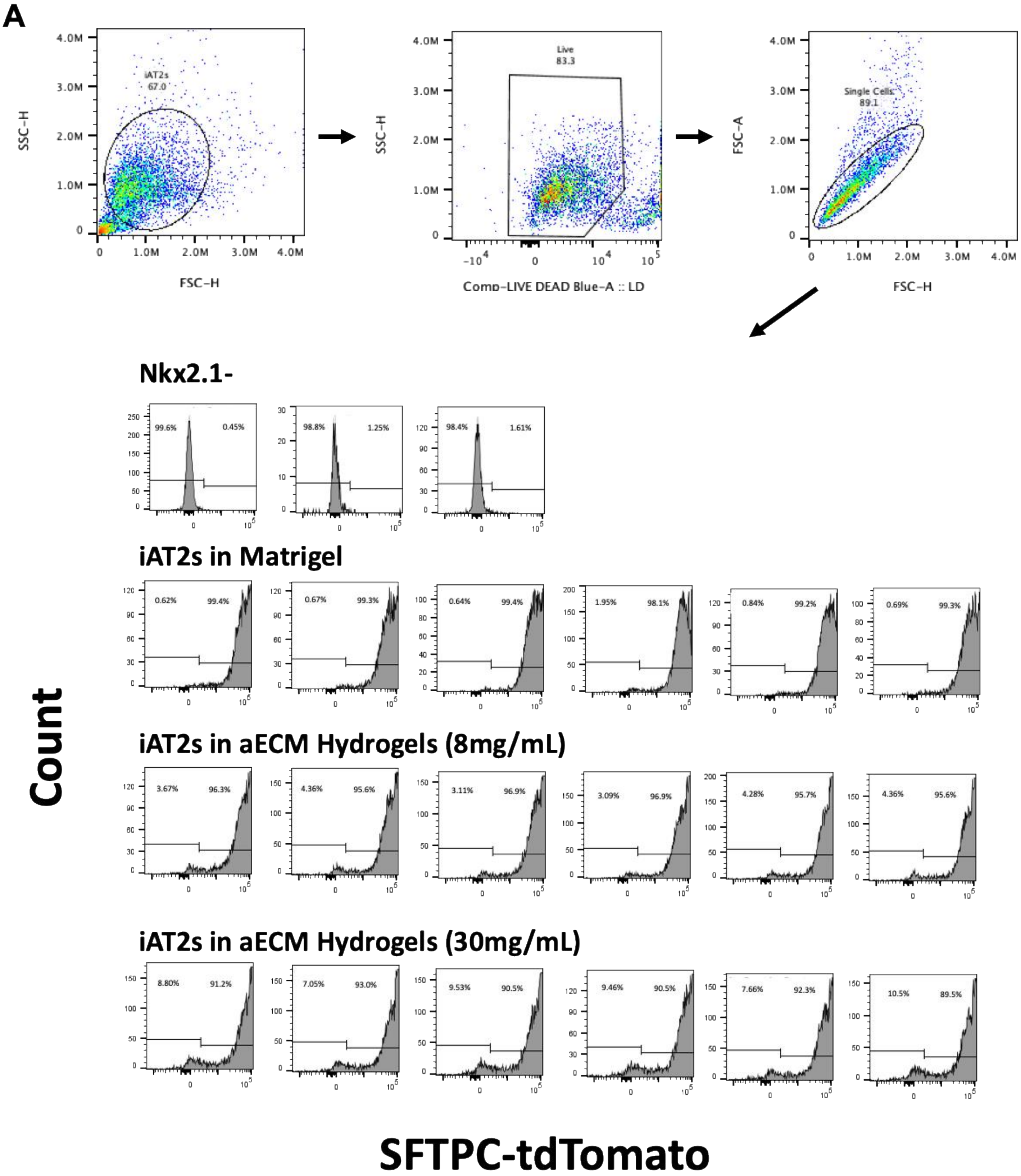

Supplemental Figure 2. Gating strategy for tdTomato expression in iAT2s. (A) Gating strategy for analyzing expression on tdTomato in live singlets of iAT2s. Nkx2.1- iPSC-derived cells from the same patient were utilized as a negative control for tdTomato expression. Raw tdTomato expression data is presented as histograms.
